# Instant termination as a novel indicator for prognosis of persistent atrial fibrillation during cryoballoon ablation: a propensity score-matched analysis

**DOI:** 10.3389/fcvm.2025.1522086

**Published:** 2025-02-28

**Authors:** Youqi Fan, Jian Ye, Xiaoya Wang, Liuguang Song, Yu Zhang, Yaping Wang

**Affiliations:** ^1^ Department of Cardiology, The Second Affiliated Hospital, School of Medicine, Zhejiang University, Hangzhou, China; ^2^ State Key Laboratory of Transvascular Implantation Devices, Hangzhou, China; ^3^ Heart Regeneration and Repair Key Laboratory of Zhejiang Province, Hangzhou, China

**Keywords:** instant termination of atrial fibrillation, cryoballoon ablation, left atrial diameter, arrhythmic recurrence, persistent atrial fibrillation

## Abstract

**Background:**

Instant atrial fibrillation termination (AFT) during radiofrequency ablation has been suggested as a predictor of prognosis in persistent atrial fibrillation (AF). However, its role in cryoballoon ablation remains unclear. This study investigated the association between AFT and recurrent atrial tachyarrhythmia in patients with persistent AF undergoing cryoballoon ablation.

**Methods:**

Patients with non-valvular, drug-resistant, persistent AF who underwent cryoballoon ablation between January 2021 and June 2023 were included and categorized based on the presence or absence of AFT. Propensity score matching (PSM) was applied to eliminate covariate imbalances. Baseline characteristics, procedural details, and clinical outcomes were compared between the groups.

**Results:**

A total of 189 patients [65.0 (59.0–71.0) years] were included. Among them, 41 experienced instant AFT, while 148 remained in AF rhythm. The baseline conditions were similar, except that patients with AFT presented significantly lower left atrial diameter (LAD). During a follow-up of 16.0 [9.1–26.9] months, the recurrence rates of arrhythmias were significantly lower in the AFT group (log-rank *P* = 0.044). Both AFT [HR: 0.298, 95% CI: (0.091–0.976), *P* = 0.035] and baseline LAD [HR: 1.079, 95% CI: (1.012–1.151), *P* = 0.021] were independent predictors of recurrence. We further assessed the prognostic value of AFT in PSM groups which showed that the recurrence rates were also significantly lower in the AFT group (log-rank *P* = 0.049).

**Conclusion:**

Instant AFT during cryoballoon ablation is associated with a reduced risk of arrhythmic recurrence in patients with persistent AF.

## Graphical Abstract

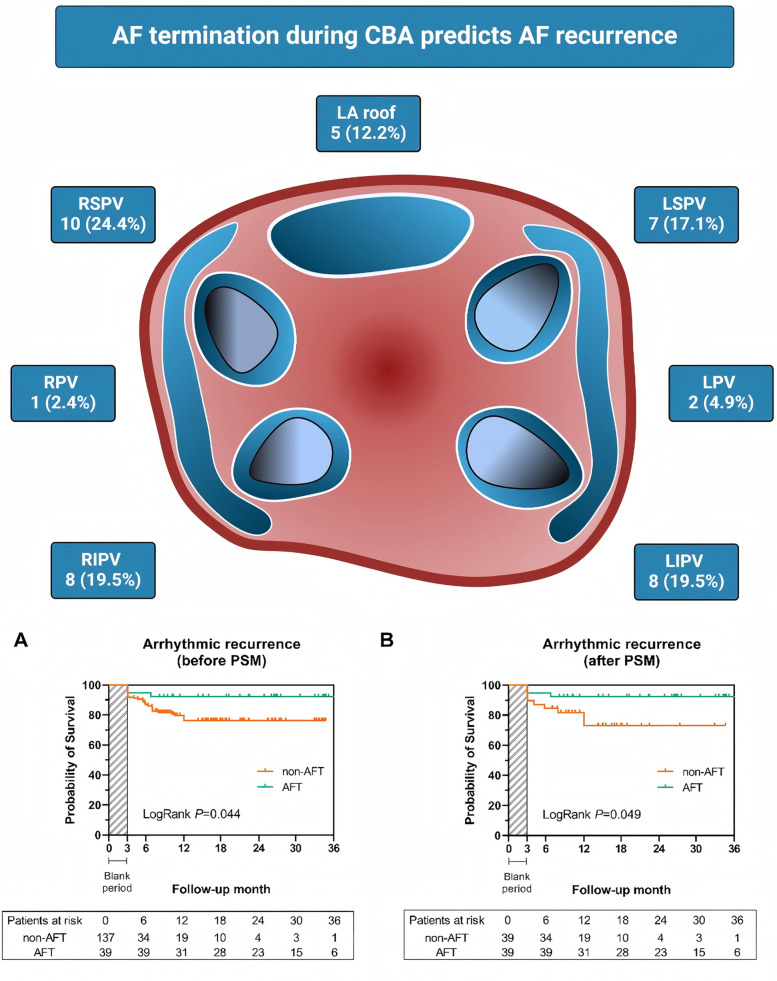

## 1 Introduction

Characterized by episodes lasting longer than 7 days, persistent atrial fibrillation (AF) is associated with a higher risk of stroke, heart failure, and mortality (1–3). Catheter ablation, a minimally invasive procedure, has emerged as a well-established treatment for persistent AF. Recent evidence indicates that catheter ablation offers superior rhythm control compared with anti-arrhythmic drug therapy (4, 5), potentially improving quality of life and reducing hospitalizations.

Instant AF termination (AFT) during ablation, leading to immediate restoration of sinus rhythm or atrial tachycardia, is considered a critical procedural endpoint of catheter ablation for persistent AF as it has been observed by some experts to predict successful long-term outcomes (6, 7), suggesting a correlation with reduced AF recurrence (8, 9). This immediate response is thought to signify effective lesion formation and adequate substrate modification, key goals in AF ablation.

However, existing studies largely focus on radiofrequency ablation, and a significant gap exists in the literature regarding the role of instant AFT in the context of cryoballoon ablation, a relatively newer technique for persistent AF management. This gap in evidence highlights a crucial area for future research, as understanding the relationship between instant AFT during cryoballoon ablation and long-term ablation success could significantly enhance procedural strategies, patient selection, and overall treatment efficacy in managing persistent AF. Therefore, our study aimed to investigate the role of instant AFT in predicting the clinical outcome of persistent AF patients who underwent cryoballoon ablation.

## 2 Materials and methods

### 2.1 Study population

**Figure 1:**
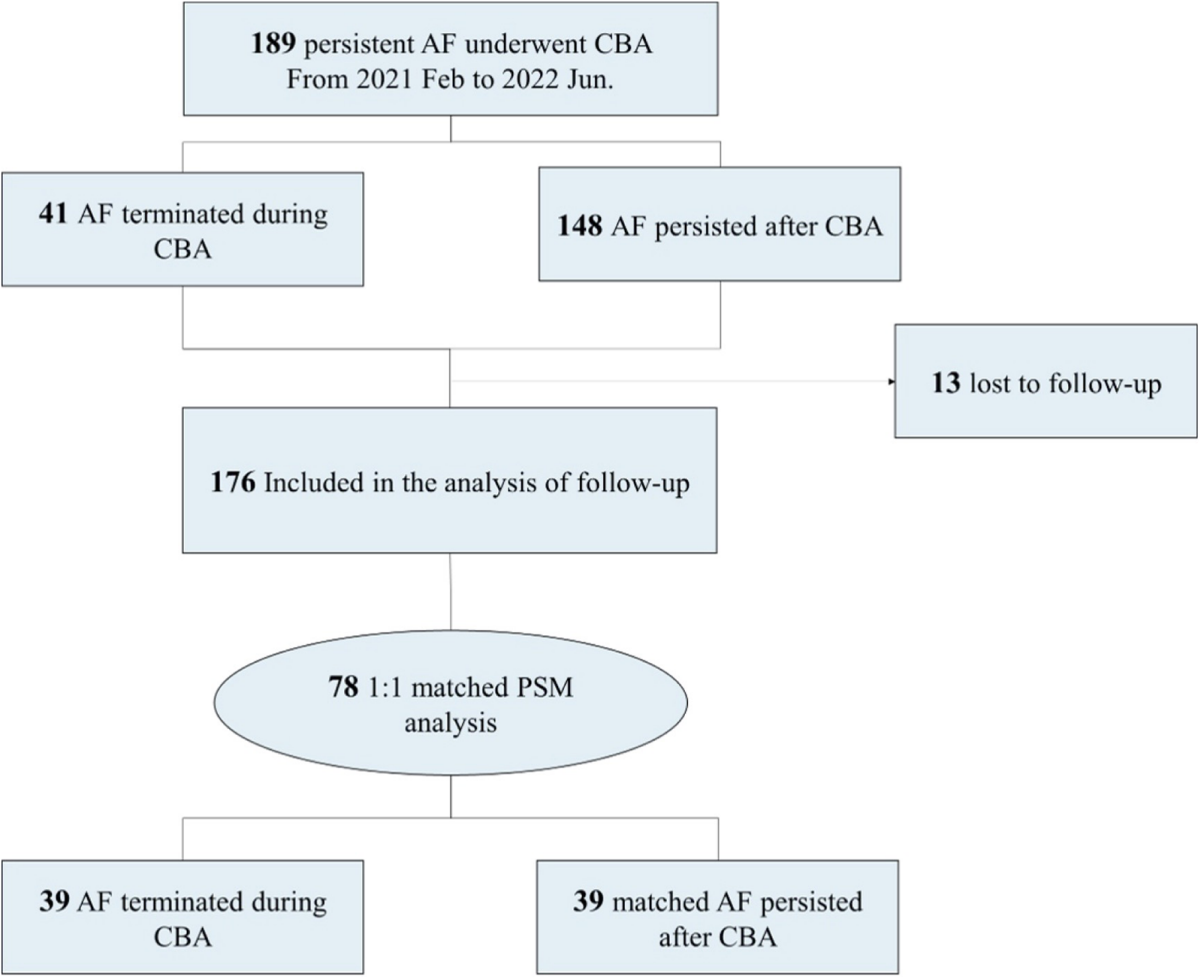
Study flowchart. AF, atrial fibrillation; CBA, cryoballoon ablation; PSM, propensity score matching.

A total of 189 patients with non-valvular, drug-resistant, persistent AF who underwent cryoballoon ablation from January 2021 to June 2022 in the Second Affiliated Hospital Zhejiang University School of Medicine were included. Among them, 41 had instant AFT during cryoballoon ablation, while 148 remained in AF rhythm after the ablation. The study flowchart is displayed in Figure 1. AF was diagnosed as absolutely irregular RR intervals and no discernible distinct *P* waves lasting longer than 30 s, as documented with a Holter monitor or electrocardiogram. AF lasting longer than 7 days or terminated by cardioversion after >7 days was defined as persistent AF. Short-term persistent AF was defined as a duration <1 year since onset, while long-standing persistent AF was defined as a duration >1 year since onset.

Patients were excluded if they had a left atrial diameter (LAD) (anterior-to-posterior) >60 mm on transthoracic echocardiography, the presence of thrombus in the left atrium or left atrial appendage (LAA) on transesophageal echocardiography or computed tomography angiography, previous history of AF ablation, acute cardiovascular events within 3 months, or malignancy with life expectancy <1 year. Since this is a retrospective observational study using de-identified data, written informed consent was not required from the patient. Our study was approved by the local institution review committee of the Second Affiliated Hospital Zhejiang University School of Medicine (approved number 2024-0463 on 16 April 2024) and complied with the precepts of the Declaration of Helsinki.

### 2.2 Cryoballoon ablation

Local anesthesia with sedation was employed for every patient. Under x-ray guidance, a single transseptal puncture was completed. A 23 or 28 mm second-generation cryoballoon (Arctic Front Advance, Medtronic, MN, USA) was advanced through a steerable sheath (FlexCath Advance, Medtronic, MN, USA) into the left atrium. Once the pulmonary vein was confirmed, the cryoballoon was further inflated and advanced to the ostium of the pulmonary vein, following angiography with the injection of contrast dye to ensure complete pulmonary vein occlusion.

The Achieve catheter (Achieve, Medtronic, MN, USA) was cannulated distally into the pulmonary vein for the measurement of electric activity. The cryoballoon ablation followed the sequence of left superior pulmonary vein (LSPV), left inferior pulmonary vein (LIPV), right superior pulmonary vein (RSPV), and right inferior pulmonary vein (RIPV). A standard 180 s freeze was adopted for each pulmonary vein including those without a recorded potential. The freezing time and duration were adjusted according to our protocol based on the time to isolation (TTI).

Generally, the freezing time was set to 150–180 s when TTI was ≤30 s. When the TTI was between 30 and 60 s, the freezing time was set to 180 s. A bonus freeze of 120 s was applied only when the TTI was >60 s. If the TTI could not be recorded, a 180 s freeze with or without a bonus freeze of 120 s (when the temperature decreased below −40℃ within 60 s after the application) was adopted.

During the freezing of the RSPV and RIPV, continuous phrenic pacing (8–10 V; pace interval, 2,000 ms) with an electrode placed in the superior vena cava was applied. Phrenic nerve palsy was monitored by observing diaphragm movement under fluoroscopy. If a decrease in diaphragm movement was detected, the freezing procedure was subsequently halted to prevent further injury.

In the event of atrial flutter or tachycardia during the ablation procedure, the operator first attempted to map and ablate the relevant sites. If termination was unsuccessful or the patient could not tolerate the procedure, cardioversion was performed. Heparin was intravenously administered throughout the procedure, with the activated clotting time carefully monitored to maintain a range of 250–300 s.

Following the ablation, the Achieve catheter was routinely employed for pulmonary vein potential mapping and pacing verification. Electrophysiology studies and atrial burst pacing were subsequently performed to confirm the isolation effect.

Instant AFT was defined as the conversion of AF to sinus rhythm during cryoballoon ablation at any of the locations, including the pulmonary vein, left atrium roof, and left and right pulmonary vein antra.

### 2.3 Periprocedural preparation

Upon admission, current and past medical histories of all patients were acquired after admission. Physical examinations were performed by experienced physicians, and routine laboratory examinations were carried out. Transthoracic echocardiography was performed for every patient. Transesophageal echocardiography or computed tomography angiography was performed 1 day prior to the procedure to assess for thrombus in the left atrium or LAA, re-evaluate the left atrial size, and identify any structural lesions.

Before the procedure, anti-arrhythmic drugs were discontinued five half-lives before the procedure. All patients received anticoagulation therapy for a minimum of 8 weeks. New oral anticoagulants (dabigatran or rivaroxaban) were commonly prescribed and discontinued 24 h before the procedure. For patients taking warfarin, ablation was only considered if the international normalized ratio was stable and within the range of 2.0–3.0, and warfarin was not withdrawn before the procedure.

### 2.4 Follow-up

Patients were scheduled for outpatient follow-up visits and 24 h Holter monitoring at 1, 3, 6, and 12 months post-procedure, and then annually thereafter. A 12-lead electrocardiogram was used to detect arrhythmic recurrence. Telephone follow-up was conducted before the scheduled visit to evaluate patients' status and ensure timely re-examination. During every outpatient visit, physical examinations were also performed, and precise medical histories were taken to evaluate post-procedural complications. Additional laboratory, radiological, or echocardiographic examinations were only performed when certain indications were presented.

The primary endpoint was the recurrence of arrhythmias, defined as episodes of AF, atrial flutter, and atrial tachycardia lasting longer than 30 s after the blanking period (3 months post-procedure), which was confirmed by Holter monitor, 12-lead electrocardiogram, or data from a previously implanted device. During the blanking period, almost all patients received anti-arrhythmic drugs. If CHA_2_DS_2_-VASc ≥2 in males or ≥3 in females, oral anticoagulants were recommended for long-term anticoagulation. The continuation of anti-arrhythmic drugs was not considered a recurrence in this study.

### 2.5 Statistical analysis

Continuous variables are described as the mean ± standard deviation. Comparisons between groups were made using a two-sample *t*-test or Mann–Whitney test, depending on the equality of variance. Categorical variables are presented as percentages (%), with *P*-values derived from *χ*^2^ tests or Fisher's exact tests (for expected frequencies lower than 5). Univariate and multivariate logistic regression analysis was adopted to assess the relationship between instant AFT and other baseline variables. Model 1 was the univariate regression model, and Model 2 adjusted for age and gender. Model 3 adjusted for age, gender, body mass index (BMI), AF type, CHA_2_DS_2_-VASc score, HAS-BLED score, LAD, left ventricular ejection fraction (LVEF), estimated glomerular filtration rate (eGFR), history of hypertension, and history of diabetic mellitus.

A Kaplan–Meier analysis with the log-rank test was adopted to evaluate the recurrence risk. Both univariate and multivariate Cox proportional hazard regression models were applied to calculate the hazard ratios for baseline characteristics. Variables of age, gender, BMI, AFT, AF type, LAD, and eGFR were included in univariate analysis (*P* < 0.10), and the prognostic factors for arrhythmic recurrence were discovered through multivariate analysis. A 1:1 propensity score matching (PSM) was carried out, using a multivariate logistic regression model including age, gender, LAD, and BMI to adjust for baseline imbalances. A two-sided *P*-value of <0.05 was considered statistically significant. SAS 9.4 software (SAS Institute Inc., Cary, NC, USA) was used to conduct the analysis.

## 3 Results

A total of 189 patients were included in the study. Among them, 41 experienced instant AFT, while 148 remained in AF rhythm after cryoballoon ablation. No significant differences in age and gender were observed between the two groups. The baseline characteristics were similar, except that patients with instant AFT had significantly higher LAD [40.4 (37.7, 43.4) mm vs. 42.2 (39.6, 46.8) mm, *P* = 0.005]. A detailed comparison of baseline characteristics is listed in Table 1.

**Table 1:** Baseline characteristics of patients with or without instant AFT.

Variable	Overall (*n* = 189)	Non-AFT (*n* = 148)	AFT (*n* = 41)	*P-*value
Age, years	65.0 (59.0, 71.0)	66.0 (59.0, 71.0)	64.0 (59.0, 69.0)	0.579
Gender (female), *n* (%)	64 (33.9)	46 (31.1)	18 (43.9)	0.125
BMI, kg/m^2^	24.9 (22.8, 27.3)	25.1 (22.9, 27.5)	24.2 (22.8, 25.9)	0.160
AF type, *n* (%)				0.483
Short-term persistent	74 (39.2)	56 (37.8)	18 (43.9)	
Long-standing persistent	115 (60.9)	92 (62.2)	23 (56.1)	
CHA_2_DS_2_-VASc	2.0 (1.0, 3.0)	2.0 (1.0, 3.0)	2.0 (1.0, 3.0)	0.784
HAS-BLED	1.0 (1.0, 2.0)	1.0 (1.0, 2.0)	1.0 (0.5, 2.0)	0.734
LAD, mm	41.9 (39.0, 45.8)	42.2 (39.6, 46.8)	40.4 (37.7, 43.4)	0.005*
LVEF, %	62.8 (55.8, 67.7)	62.8 (55.2, 67.6)	62.5 (57.2, 68.7)	0.382
eGFR, ml/min/1.73 m^2^	94.3 (79.3, 113.9)	94.3 (78.9, 112.0)	94.3 (80.3, 120.6)	0.393
Hb, g/L	144.0 (132.0, 154.0)	144.0 (130.5, 156.0)	142.0 (134.0, 148.0)	0.559
CAD, *n* (%)	33 (17.5)	23 (15.5)	10 (24.4)	0.244
Hypertension, *n* (%)	118 (62.4)	95 (64.2)	23 (56.1)	0.366
SHD, *n* (%)	11 (5.8)	9 (6.1)	2 (4.9)	1.000
Diabetic mellitus, *n* (%)	34 (18.0)	24 (16.2)	10 (24.4)	0.253
CKD, *n* (%)	3 (1.6)	2 (1.4)	1 (2.4)	0.522
COPD, *n* (%)	6 (3.2)	6 (4.1)	0	0.420

Continuous variables are presented as median with interquartile range. Categorical variables are presented as frequencies and percentage (%). The asterisk (*) indicates a significant *P*-value. AF, atrial fibrillation; AFT, atrial fibrillation termination; BMI, body mass index; CAD, coronary artery disease; CKD, chronic kidney disease; COPD, chronic obstructive pulmonary disease; eGFR, estimated glomerular filtration rate; LAD, left atrial diameter; LVEF, left ventricular ejection fraction; SHD, structural heart disease.

For procedural information, AFT occurred in 41 patients out of 189 patients who underwent cryoballoon ablation, accounting for 21.6% of the study population. The highest incidence of AFT was observed during ablation of RSPV (24.4%), followed by LIPV (19.5%), RIPV (19.5%), and LSPV (17.1%). Moreover, eight cases of AFT occurred during additional pulmonary vein ablation: five (12.2%) cases at the left atrial roof, two (4.9%) cases at the left pulmonary vein (LPV) antrum, and one (2.4%) case at the right pulmonary vein (RPV) antrum.

Notably, pulmonary vein potential was more frequently recorded in patients with instant AFT, especially at RSPV [33 (80.5%) vs. 91 (61.5%), *P* = 0.023]. Furthermore, ablation tended to be more moderate in the instant AFT group, with fewer patients undergoing additional pulmonary vein ablation, particularly at the left atrial roof [29 (70.7%) vs. 125 (84.5%), *P* = 0.045]. Ablation duration was significantly shorter for LIPV and RIPV, while complication rates were comparable between the two groups. Detailed results are presented in Table 2.

**Table 2:** Procedural details of patients with or without instant AFT.

Variable	Overall (*n* = 189)	Non-AFT (*n* = 148)	AFT (*n* = 41)	*P*-value
AFT				
AFT site, *n* (%)				
LSPV	—	—	7 (17.1)	—
LIPV	—	—	8 (19.5)	—
RSPV	—	—	10 (24.4)	—
RIPV	—	—	8 (19.5)	—
left atrial roof			5 (12.2)	
LPV antrum			2 (4.9)	
RPV antrum			1 (2.4)	
Time to AFT, s	—	—	270.0 (180.0, 450.0)	—
AFT nadir, ℃	—	—	−48.0 (−53.0, −42.0)	—
Additional PV sites, *n* (%)				
Left atrial roof	154 (81.5)	125 (84.5)	29 (70.7)	0.045*
LPV antrum	98 (51.9)	73 (49.3)	25 (61.0)	0.186
RPV antrum	127 (67.2)	98 (66.2)	29 (70.7)	0.586
SVC	2 (1.1)	2 (1.4)	0	1.000
Left common PV	1 (0.5)	1 (0.7)	0	1.000
Ablation duration, min	28.8 (25.5, 32.1)	28.7 (26.3, 32.1)	29.0 (24.6, 31.5)	0.378
LSPV	180.0 (180.0, 300.0)	180.0 (180.0, 300.0)	180.0 (180.0, 330.0)	0.070
LIPV	300.0 (270.0, 300.0)	300.0 (270.0, 300.0)	180.0 (180.0, 273.5)	<0.001*
RSPV	180.0 (150.0, 270.0)	180.0 (125.5, 270.0)	180.0 (120.0, 270.0)	0.231
RIPV	300.0 (245.0, 390.0)	300.0 (270.0, 390.0)	270.0 (180.0, 360.0)	0.029*
Left atrial roof	600.0 (480.0, 660.0)	600.0 (480.0, 720.0)	540.0 (420.0, 660.0)	0.108
LPV antrum	155.0 (120.0, 270.0)	150.0 (120.0, 240.0)	240.0 (120.0, 280.0)	0.069
RPV antrum	120.0 (120.0, 240.0)	120.0 (120.0, 180.0)	120.0 (120.0, 240.0)	0.109
Nadir temperature, ℃				
LSPV	−51.0 (−55.0, −46.5)	−51.0 (−55.0, −46.0)	−51.0 (−52.0, −47.0)	0.282
LIPV	−45.0 (−50.0, −42.0)	−46.0 (−51.0, −42.0)	−44.5 (−48.5, −40.0)	0.231
RSPV	−54.0 (−56.0, −48.0)	−53.0 (−56.0, −47.0)	−55.0 (−56.0, −51.0)	0.332
RIPV	−49.5 (−55.0, −44.0)	−49.0 (−55.0, −44.0)	−50.0 (−55.0, −43.0)	0.924
Left atrial roof	−45.0 (−49.0, −42.0)	−45.0 (−48.0, −42.0)	−45.0 (−49.0, −41.0)	0.873
LPV antrum	−40.0 (−43.0, −35.0)	−39.0 (−43.0, −35.0)	−41.0 (−45.0, −38.0)	0.129
RPV antrum	−44.0 (−50.0, −37.0)	−44.0 (−50.0, −36.0)	−45.0 (−52.0, −41.0)	0.136
PV potential recorded, *n* (%)				
LSPV	122 (64.6)	91 (61.5)	31 (75.6)	0.094
LIPV	131 (69.3)	101 (68.2)	30 (73.2)	0.545
RSPV	124 (80.5)	91 (61.5)	33 (80.5)	0.023*
RIPV	110 (64.3)	81 (62.3)	29 (70.7)	0.326
TTI, s				
LSPV	43.0 (35.0, 54.0)	43.0 (35.0, 54.0)	43.0 (33.0, 55.0)	0.874
LIPV	28.0 (18.0, 39.0)	28.0 (19.0, 37.0)	26.5 (18.0, 40.0)	0.555
RSPV	33.0 (25.0, 43.0)	34.0 (25.0, 43.0)	32.0 (25.0, 47.0)	0.802
RIPV	32.0 (22.0, 49.0)	32.0 (25.0, 47.0)	36.0 (20.0, 60.0)	0.916
Complications, *n* (%)				
Phrenic nerve palsy	1 (0.5)	1 (0.7)	0	1.000

Continuous variables are presented as median with interquartile range. Categorical variables are presented as frequencies and percentage (%). The asterisk (*) indicates a significant *P*-value. AFT, atrial fibrillation termination; LIPV, left inferior pulmonary vein; LPV, left pulmonary vein; LSPV, left superior pulmonary vein; PV, pulmonary vein; RIPV, right inferior pulmonary vein; RPV, right pulmonary vein; RSPV, right superior pulmonary vein; SVC, superior vein cava; TTI, time to isolation.

**Figure 2:**
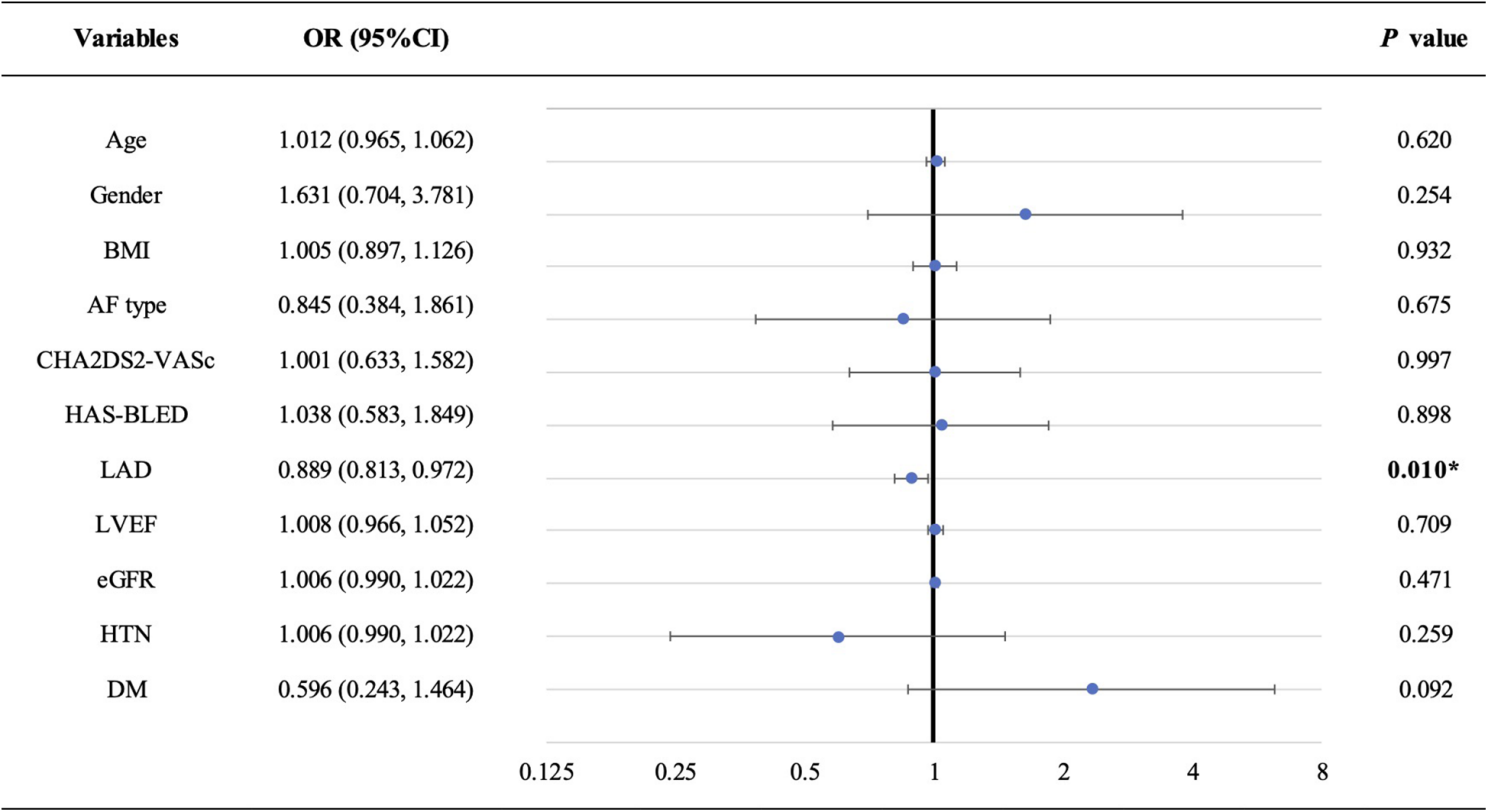
Forest plot of multivariate logistic regression analysis. LAD is the only factor that is significantly associated with AFT (adjusted OR: 0.889, 95% CI: 0.813–0.972; *P* = 0.010). AF, atrial fibrillation; AFT, atrial fibrillation termination; BMI, body mass index; DM, diabetic mellitus; eGFR, estimated glomerular filtration rate; HTN, hypertension; LAD, left atrial diameter; LVEF, left ventricular ejection fraction.

To investigate the relationship between AFT and baseline characteristics, we conducted a logistic regression analysis (Figure 2 and Supplementary Table 1). Our findings revealed that LAD was the only significant factor associated with AFT, with an adjusted OR (95% CI) of 0.889 (0.813, 0.972) with *P* = 0.010, suggesting that a larger LAD is associated with a lower frequency of AFT.

**Figure 3:**
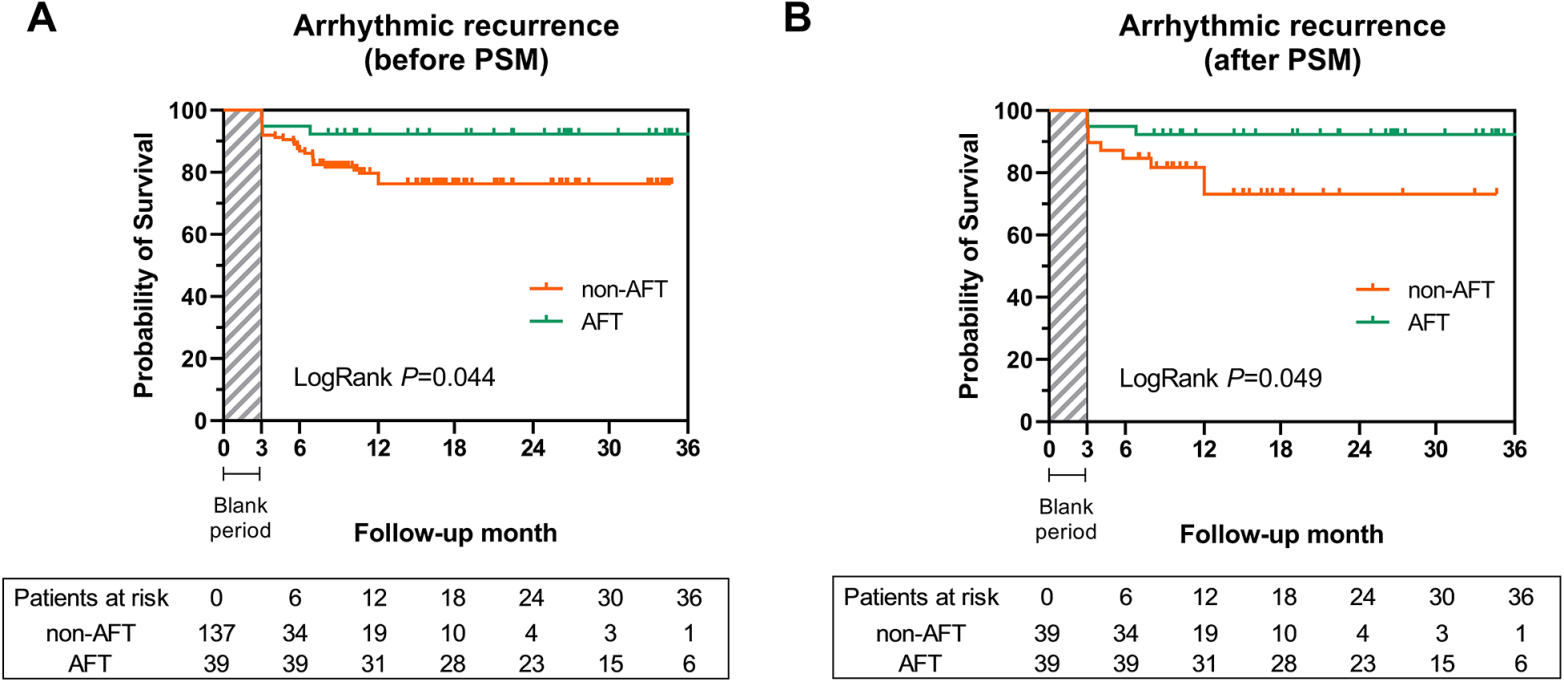
Kaplan-Meier curve for the risk of arrhythmic recurrence among the AFT group and non-AFT group before and after PSM. **(A)** Cumulative survival free from arrhythmic recurrence was significantly higher in the AFT group before PSM (log-rank *P* = 0.044). **(B)** The AFT group also presented a higher survival rate free from arrhythmic recurrence after PSM (log-rank *P* = 0.049). AF, atrial fibrillation; AFT, atrial fibrillation termination; PSM, propensity score matching.

Through a follow-up of 16.0 (9.1, 26.9) months, 13 (6.9%) patients were lost due to changes in their contact information or their residence being too far from the hospital, and 1 patient died in a car accident. Most follow-up events were similar between the two groups, including a non-significant higher occurrence of AF redo ablation in the non-AFT group. Notably, 33 cases (18.5%) of recurrent arrhythmia were observed (shown in Table 3), and recurrence rates differed significantly between the AFT and non-AFT groups (Figure 3A, 7.7% vs. 21.9%, log-rank *P* = 0.044). A Cox proportional hazard model showed that both AFT [0.298 (0.091, 0.976), *P* = 0.035] and baseline LAD [1.079 (1.012, 1.151), *P* = 0.021] could independently predict the risk of recurrence (Table 4).

**Table 3:** Detailed follow-up events of patients with or without instant AFT.

Events	Overall (*n* = 176)	Non-AFT (*n* = 137)	AFT (*n* = 39)	*P*-value
Follow-up events after ablation, *n* (%)				
Recurrence	33 (18.5)	30 (21.9)	3 (7.7)	0.044*
Death	1 (0.6)	1 (0.7)	0	1.000
Redo ablation	9 (5.1)	9 (6.6)	0	0.218
LAAC	1 (0.6)	1 (0.7)	0	1.000
Bradycardia	2 (1.1)	1 (0.7)	1 (0.7)	0.923
AMI	1 (0.6)	1 (0.7)	0	1.000
Anti-arrhythmic drugs during the blanking period, *n* (%)				
I/III	154 (87.5)	126 (92.0)	28 (71.8)	0.002*
II	80 (45.5)	60 (43.8)	20 (51.3)	0.407
Anti-arrhythmic drugs after the blanking period, *n* (%)	6 (3.4)	6 (4.4)	0	0.407

Continuous variables are presented as median with interquartile range. Categorical variables are presented as frequencies and percentage (%). The asterisk (*) indicates a significant *P*-value. AF, atrial fibrillation; AFT, atrial fibrillation termination; AMI, acute myocardial infarction; LAAC, left atrial appendage closure.

**Table 4:** Cox proportional hazard model for the arrhythmic recurrence.

Variables	Univariate		Multivariate	
	HR (95% CI)	*P*-value	HR (95% CI)	*P*-value
Age	1.012 (0.974–1.052)	0.529	1.016 (0.977–1.057)	0.415
Gender	1.194 (0.594–2.401)	0.618	1.058 (0.482–2.236)	0.886
BMI	1.018 (0.931–1.115)	0.690	0.985 (0.899–1.079)	0.747
AFT	0.317 (0.097–1.038)	0.058	0.298 (0.091–0.976)	0.035*
AF type	1.930 (0.871–4.280)	0.106	1.734 (0.775–3.880)	0.181
CHA_2_DS_2_-VASc	1.221 (0.910–1.638)	0.183	—	—
HAS-BLED	1.117 (0.775–1.612)	0.553	—	—
LAD	1.074 (1.014–1.137)	0.015*	1.079 (1.012–1.151)	0.021*
LVEF	0.993 (0.959–1.030)	0.718	—	—
eGFR	0.991 (0.979–1.002)	0.118	0.991 (0.977–1.006)	0.233
Hypertension	0.999 (0.461–2.165)	0.998	—	—
Diabetic mellitus	0.879 (0.334–2.317)	0.795	—	—
Additional PV sites				
Left atrial roof	0.752 (0.319–1.771)	0.514	—	—
LPV antrum	0.732 (0.346–1.549)	0.415	—	—
RPV antrum	0.715 (0.338–1.515)	0.382	—	—
Ablation duration				
LSPV	0.999 (0.996–1.002)	0.503	—	—
LIPV	1.003 (0.999–1.007)	0.110	—	—
RSPV	0.997 (0.992–1.002)	0.261	—	—
RIPV	1.002 (1.000–1.003)	0.111	—	—
Left atrial roof	1.001 (0.999–1.003)	0.373	—	—
LPV antrum	1.003 (1.000–1.007)	0.059	—	—
RPV antrum	0.999 (0.994–1.004)	0.661	—	—

The asterisk (*) indicates a significant *P*-value. AF, atrial fibrillation; AFT, atrial fibrillation termination; BMI, body mass index; eGFR, estimated glomerular filtration rate; LAD, left atrial diameter; LIPV, left inferior pulmonary vein; LPV, left pulmonary vein; LSPV, left superior pulmonary vein; LVEF, left ventricular ejection fraction; PV, pulmonary vein; RIPV, right inferior pulmonary vein; RPV, right pulmonary vein; RSPV, right superior pulmonary vein.

Given the baseline differences in LAD, we further assessed the prognostic value of AFT using the 1:1 PSM analysis. After matching, 39 patients with AFT were compared with 39 patients without AFT, and baseline characteristics (including LAD, Table 5) and procedural information (Supplementary Table 2) were comparable. Survival analysis demonstrated that the recurrence risk in the AFT-matched group remained significantly lower than in the non-AFT-matched group (Figure 3B, log-rank *P* = 0.049).

**Table 5:** Comparison of baseline characteristics between PSM groups.

Variable	Overall (*n* = 78)	Non-AFT-matched (*n* = 39)	AFT-matched (*n* = 39)	*P*-value
Age, years	65.0 (59.0, 71.0)	66.0 (60.0, 71.0)	64.0 (59.0, 72.0)	0.501
Gender (female), *n* (%)	32 (41.0)	14 (35.9)	18 (46.2)	0.357
BMI, kg/m^2^	24.3 (22.5, 26.1)	24.7 (22.4, 26.1)	24.0 (22.5, 25.9)	0.672
AF type, *n* (%)				0.241
Short-term persistent	29 (37.2)	12 (30.8)	17 (43.6)	
Long-standing persistent	49 (62.8)	27 (69.2)	22 (56.4)	
CHA_2_DS_2_-VASc	2.0 (1.0, 3.0)	2.0 (1.0, 3.0)	2.0 (1.0, 3.0)	0.850
HAS-BLED	1.0 (1.0, 2.0)	1.0 (1.0, 2.0)	1.0 (0.0, 2.0)	0.235
LAD, mm	40.1 (37.9, 43.4)	40.1 (38.2, 43.4)	40.1 (37.5, 43.4)	0.784
LVEF, %	63.8 (57.3, 68.6)	64.2 (57.6, 68.6)	61.9 (57.0, 68.7)	0.819
eGFR, ml/min/1.73 m^2^	95.3 (78.1, 113.2)	96.1 (76.6, 109.9)	94.1 (79.5, 120.6)	0.647
Hb, g/L	142.5 (132.0, 151.0)	145.0 (124.0, 160.0)	142.0 (133.0, 148.0)	0.323
CAD, *n* (%)	17 (21.8)	7 (18.0)	10 (25.6)	0.411
Hypertension, *n* (%)	30 (38.5)	12 (30.8)	19 (46.2)	0.163
SHD, *n* (%)	4 (5.1)	2 (5.1)	2 (5.1)	1.000
Diabetic mellitus, *n* (%)	17 (21.8)	7 (18.0)	10 (25.6)	0.411
CKD, *n* (%)	1 (1.3)	0	1 (2.6)	1.000
COPD, *n* (%)	3 (3.9)	3 (7.7)	0	0.239

Continuous variables are presented as median with interquartile range. Categorical variables are presented as frequencies and percentage (%). The asterisk (*) indicates a significant *P*-value. AF, atrial fibrillation; AFT, atrial fibrillation termination; BMI, body mass index; CAD, coronary artery disease; CKD, chronic kidney disease; COPD, chronic obstructive pulmonary disease; eGFR, estimated glomerular filtration rate; LAD, left atrial diameter; LVEF, left ventricular ejection fraction; SHD, structural heart disease.

## 4 Discussion

### 4.1 Ablation strategies for persistent AF

Pulmonary vein isolation (PVI) remains the cornerstone of catheter ablation for persistent AF (10). A meta-analysis of 14 studies showed a 1-year arrhythmia-free survival rate of 66.7% for PVI alone (11). However, whether PVI alone is sufficient for persistent AF remains debated. The STAR AF II trial, which included 589 patients, found no significant difference in the risk of AF recurrence after 18 months between those who underwent PVI alone (59% free of recurrence) and those who had PVI plus additional ablation strategies (49% for CFAE, 46% for linear ablation) (12). Similarly, other studies have demonstrated that adding extra ablation steps did not significantly improve outcomes (13–17).

While new ablation techniques continue to emerge, the complex mechanisms underlying persistent AF, along with potential complications from longer procedures, suggest that further research is needed to define optimal ablation targets and protocols.

### 4.2 Procedural endpoints of cryoballoon ablation

PVI and TTI are both established endpoints for cryoballoon ablation. PVI has been proven to improve outcomes in persistent AF (18). However, pulmonary vein reconnection can occur after PVI, leading to recurrent AF or atrial tachycardia (19–21). The adenosine test is performed to verify the presence or recovery of pulmonary vein block and assess the need for further ablation. Mean TTI has been identified as an independent predictor of AF recurrence (HR: 1.008; 95% CI: 1.002–1.014; *P* = 0.010) (22), with a TTI ≤60 s associated with more durable PVI (23). Nonetheless, TTI is not always measurable during procedures, limiting its utility.

Instant AFT as an endpoint offers the advantage of guiding procedural strategy. Our study suggests that the occurrence of AFT during cryoballoon ablation is associated with a lower recurrence risk, potentially reducing the need for extensive ablations. Singh et al. also proposed that PVI alone may be optimal for patients who experience AFT (24). In contrast, the absence of AFT may indicate incomplete transmural lesions or extra initiating and maintenance mechanisms; thus, further ablation is required.

### 4.3 The mechanism of instant AFT

Santangeli et al. reported that nearly 90% of persistent AF originated from the pulmonary vein (25). In our study, approximately 80% of AFT cases occurred during PVI, suggesting a potential connection between AFT sites and AF onset.

We observed that LAD independently predicted the occurrence of AFT, with smaller LAD values in patients who experienced AFT, consistent with other studies (7, 9, 24). In addition to left atrial enlargement, a longer duration of AF and more severe AF classification are associated with a lower incidence of AFT and a poorer prognosis for PVI (26, 27). We hypothesize that the site where AFT occurs may act as an AF driver, contributing to both the initiation and perpetuation of AF. In the early stages of AF, ectopic activity in the pulmonary veins serves as the primary trigger. However, as AF progresses, atrial structural remodeling, particularly tissue fibrosis, results in extrapulmonary vein triggers and conduction abnormalities that sustain AF (28). Consequently, during early AF, pulmonary vein triggers predominate, and PVI can effectively eliminate these triggers, leading to AFT and favorable clinical outcomes. In later stages of AF, characterized by larger LAD, more severe AF, and longer AF duration, the underlying mechanisms become more intricate, and ectopic activity of pulmonary veins is no longer the dominant factor, resulting in a reduced incidence of AFT and less benefit from PVI.

Thus, we propose two reasons why AFT is linked to a lower risk of arrhythmic recurrence. Firstly, AFT indicates the successful elimination of AF trigger foci, making additional ablations unnecessary. Secondly, patients with AFT tend to have less advanced AF, which is associated with simpler mechanisms and better outcomes following PVI.

### 4.4 Limitations

There are several limitations to this study. First, it is a single-center retrospective study with a relatively small sample size, particularly for patients with instant AFT. Although we used multivariable Cox regression and 1:1 PSM to strengthen our conclusions, bias due to the small sample size is inevitable. A well-designed multicenter study with a larger sample size and longer follow-up period is needed to provide stronger evidence and identify additional factors associated with AFT.

Second, the population in our study was relatively younger and healthier, with a maximum age of 71 years, good left ventricular ejection fraction, and small atria, which may limit the generalizability of our findings. However, our results have clinical significance, as approximately 60% of patients had long-standing persistent AF, which is typically more challenging to treat. This suggests that AFT could be a useful prognostic indicator for patients with long-standing persistent AF undergoing cryoballoon ablation. Future studies should explore the predictive value of AFT in this subgroup.

Third, the 24 h Holter monitoring may not fully capture arrhythmia episodes, particularly if patients experience silent AF, potentially underestimating the actual recurrence rate.

Finally, instant AFT can be followed by various termination patterns, such as sinus rhythm or atrial tachycardia, each indicating a distinct underlying mechanism and suggesting different clinical outcomes, while our study focused solely on the conversion from AF to sinus rhythm.

## 5 Conclusion

Baseline LAD was strongly correlated with instant AFT, and both factors independently predicted the recurrence of atrial tachyarrhythmia. However, AFT itself was associated with a lower risk of recurrence in patients with persistent AF, regardless of LAD. These findings suggest that instant AFT may serve as a novel prognostic indicator for the outcomes of cryoballoon ablation.

## Data Availability

The raw data supporting the conclusions of this article will be made available by the authors, without undue reservation.
