# Supplementary material for: Instant termination as a novel indicator for prognosis of persistent atrial fibrillation during cryoballoon ablation: a propensity score-matched analysis
Source: Front Cardiovasc Med. 2025 Feb 28;12:1522086. doi: 10.3389/fcvm.2025.1522086 (PMC11906448; doi:10.3389/fcvm.2025.1522086)
Supplement: Supplementary file 1 [file Table1.pdf]

Supplementary Material

1 Supplementary Tables

Supplementary table 1 Logistic regression model for AFT.

Variables	Model 1		Model 2		Model 3	
	OR (95%CI)	*P* value	OR (95%CI)	*P* value	OR (95%CI)	*P* value
Age	0.994 (0.957, 1.031)	0.735	0.994 (0.957, 1.033)	0.766	1.012 (0.965, 1.062)	0.620
Gender	1.736 (0.855, 3.524)	0.127	1.893 (0.917, 3.905)	0.084	1.631 (0.704, 3.781)	0.254
BMI	0.939 (0.852, 1.034)	0.199	0.937 (0.850, 1.033)	0.191	1.005 (0.897, 1.126)	0.932
AF type	0.778 (0.386, 1.567)	0.482	0.750 (0.370, 1.522)	0.426	0.845 (0.384, 1.861)	0.675
CHA_2DS_2-VASc	1.063 (0.811, 1.393)	0.659	1.088 (0.826, 1.433)	0.547	1.001 (0.633, 1.582)	0.997
HAS-BLED	0.927 (0.649, 1.324)	0.677	0.938 (0.653, 1.349)	0.730	1.038 (0.583, 1.849)	0.898
LAD	0.916 (0.852, 0.985)	**0.017***	0.915 (0.851, 0.984)	**0.016***	0.889 (0.813, 0.972)	**0.010***
LVEF	1.026 (0.988, 1.065)	0.189	1.026 (0.988, 1.066)	0.188	1.008 (0.966, 1.052)	0.709
eGFR	1.010 (0.996, 1.023)	0.170	1.008 (0.994, 1.023)	0.262	1.006 (0.990, 1.022)	0.471
Hypertension	0.713 (0.353, 1.439)	0.345	0.699 (0.342, 1.426)	0.325	0.596 (0.243, 1.464)	0.259

Diabetic mellitus	1.667 (0.722, 3.846)	0.231	1.578 (0.678, 3.672)	0.290	2.331 (0.872, 6.233)	0.092

Asterisk (*) indicates significant P-value. AF: atrial fibrillation; AFT: atrial fibrillation termination; BMI: body mass index; eGFR: estimated glomerular filtration rate; LAD: left atrial diameter; LVEF: left ventricular ejection fraction.

Supplementary table 2. Comparison of baseline characteristics between PSM matched groups.

**Variable**	**Overall** (n=78)	**Non-AFT$_{matched}$** (n=39)	**AFT$_{matched}$** (n=39)	***P* value**
**Extra PV sites, n (%)**				
Left atrial roof	64 (82.1)	36 (92.3)	28 (71.8)	0.018
LPV antrum	45 (57.7)	20 (51.3)	25 (64.1)	0.252
RPV antrum	54 (69.2)	26 (66.7)	28 (71.8)	0.624
SVC				
Ablation duration, min	28.6 (25.2, 31.3)	28.4 (25.2, 31.0)	28.8 (24.5, 33.5)	0.942
LSPV	180.0 (180.0, 300.0)	180.0 (180.0, 250.5)	180.0 (180.0, 330.0)	0.095
LIPV	270.0 (180.0, 300.0)	300.0 (270.0, 300.0)	180.0 (180.0, 270.0)	<0.001
RSPV	180.0 (150.0, 270.0)	170.0 (120.0, 263.0)	180.0 (160.0, 270.0)	0.056
RIPV	271.0 (240.0, 360.0)	300.0 (270.0, 390.0)	270.0 (180.0, 360.0)	0.134
Left atrial roof	540.0 (480.0, 660.0)	578.0 (480.0, 675.0)	540.0 (390.0, 660.0)	0.369
LPV antrum	180.0 (120.0, 270.0)	135.0 (120.0, 270.0)	240.0 (120.0, 280.0)	0.082

RPV antrum	120.0 (120.0, 240.0)	120.0 (120.0, 180.0)	195.0 (120.0, 240.0)	0.074
**Nadir temperature, °C**				
LSPV	-51.0 (-55.0, -47.0)	-52.5 (-56.0, -48.5)	-51.0 (-53.0, -47.0)	0.091
LIPV	-44.0 (-49.0, -41.0)	-44.0 (-51.0, -41.0)	-44.5 (-48.0, -40.0)	0.700
RSPV	-54.0 (-56.0, -51.0)	-54.0 (-57.5, -47.5)	-55.0 (-56.0, -51.0)	0.751
RIPV	-50.5 (-55.0, -44.0)	-51.0 (-55.0, -45.0)	-50.0 (-55.0, -43.0)	0.685
Left atrial roof	-45.5 (-49.5, -41.0)	-46.0 (-50.0, -41.0)	-45.0 (-49.5, -41.0)	0.951
LPV antrum	-40.0 (-45.0, -36.0)	-39.0 (-43.5, -34.0)	-41.0 (-45.0, -38.0)	0.160
RPV antrum	-44.0 (-51.0, -38.0)	-43.0 (-49.0, -38.0)	-45.0 (-51.5, -40.5)	0.776
**TTI recorded, n (%)**				
LSPV	61 (78.2)	30 (76.9)	31 (79.5)	0.784
LIPV	61 (78.2)	31 (79.5)	30 (76.9)	0.784
RSPV	63 (80.7)	31 (79.5)	32 (82.1)	0.774
RIPV	53 (70.7)	25 (69.4)	28 (71.8)	0.823
**TTI, sec**				
LSPV	48.0 (35.0, 55.0)	50.0 (36.0, 60.0)	43.0 (33.0, 55.0)	0.394
LIPV	26.0 (18.0, 38.0)	25.0 (17.0, 37.0)	26.5 (18.0, 40.0)	0.897
RSPV	36.0 (25.0, 44.0)	37.0 (28.0, 43.0)	31.0 (24.0, 47.0)	0.393
RIPV	31.0 (20.0, 48.0)	30.0 (20.0, 42.0)	34.0 (20.0, 60.0)	0.570
**Complications, n (%)**				

Phrenic nerve palsy	0	0	0	1.000

Continuous variables are presented as median with interquartile range. Categorical variables are presented as frequencies and percentage (%). Asterisk (*) indicates significant P-value. AFT: atrial fibrillation termination; LIPV: left inferior pulmonary vein; LPV: left pulmonary vein; LSPV: left superior pulmonary vein; RIPV: right inferior pulmonary vein; RPV: right pulmonary vein; RSPV: right superior pulmonary vein; SVC: superior vein cava; TTI: time to isolation.
